# Locked Nucleic Acid Hydrolysis Probes for the Specific Identification of Probiotic Strains *Bifidobacterium animalis* subsp. *lactis* DSM 15954 and Bi-07™

**DOI:** 10.3389/fmicb.2021.801795

**Published:** 2021-12-23

**Authors:** Hanan R. Shehata, Anthony Kiefer, Wesley Morovic, Steven G. Newmaster

**Affiliations:** ^1^Natural Health Product Research Alliance, College of Biological Science, University of Guelph, Guelph, ON, Canada; ^2^Department of Microbiology, Faculty of Pharmacy, Mansoura University, Mansoura, Egypt; ^3^IFF Health & Biosciences, International Flavors and Fragrances, Inc., Madison, WI, United States

**Keywords:** real-time PCR, probe-based, strain-specific, locked nucleic acid probe, *Bifidobacterium animalis* subsp. *lactis*, probiotics, authentication

## Abstract

Probiotic health benefits are now well-recognized to be strain specific. Probiotic strain characterization and identification is thus important in clinical research and in the probiotic industry. This is becoming especially important with reports of probiotic products failing to meet the declared strain content, potentially compromising their efficacy. Availability of reliable identification methods is essential for strain authentication during discovery, evaluation and commercialization of a probiotic strain. This study aims to develop identification methods for strains *Bifidobacterium animalis* subsp. *lactis* DSM 15954 and Bi-07 (Bi-07™) based on real-time PCR, targeting single nucleotide polymorphisms (SNPs). The SNPs were targeted by PCR assays with locked nucleic acid (LNA) probes, which is a novel application in probiotic identification. The assays were then validated following the guidelines for validating qualitative real-time PCR assays. Each assay was evaluated for specificity against 22 non-target strains including closely related *Bifidobacterium animalis* subsp. *lactis* strains and were found to achieve 100% true positive and 0% false positive rates. To determine reaction sensitivity and efficiency, three standard curves were established for each strain. Reaction efficiency values were 86, 91, and 90% (R square values > 0.99), and 87, 84, and 86% (R square values > 0.98) for *B. animalis* subsp. *lactis* DSM 15954 and Bi-07 assays, respectively. The limit of detection (LOD) was 5.0 picograms and 0.5 picograms of DNA for DSM 15954 and Bi-07 assays, respectively. Each assay was evaluated for accuracy using five samples tested at three different DNA concentrations and both assays proved to be highly repeatable and reproducible. Standard deviation of Cq values between two replicates was always below 1.38 and below 1.68 for DSM 15954 and Bi-07 assays, respectively. The assays proved to be applicable to mono-strain and multi-strain samples as well as for samples in various matrices of foods or dietary supplement ingredients. Overall, the methods demonstrated high specificity, sensitivity, efficiency and precision and broad applicability to sample, matrix and machine types. These methods facilitate strain level identification of the highly monophyletic strains *B. animalis* subsp. *lactis* DSM 15954 and Bi-07 to ensure probiotic efficacy and provide a strategy to identify other closely related probiotics organisms.

## Introduction

Recent years have witnessed a significant increase in scientific investigations of probiotics with over 20,000 publications as of February 2019 (Reid et al., 2019). There has also been a rapid increase in the global probiotic market size which was valued at USD 48.88 billion in 2019 and expected to reach USD 94.48 billion by end of 2027 (Fortune-Business-Insights, 2020). The rapid growth in scientific research and in the market size of probiotics was accompanied by reports on non-compliance and fraud in probiotic products (Morovic et al., 2016; Patro et al., 2016; Kolaček et al., 2017; Shehata and Newmaster, 2020b,c). A major form of non-compliance in probiotic products is failure of products to meet label claims of strain contents which can be encountered as substituted strains, missing strains or presence of undeclared strains (Shehata and Newmaster, 2020b).

Correct probiotic characterization was identified as one of the criteria to qualify a microorganism as probiotic (Binda et al., 2020). Relevant to correct probiotic characterization is proper strain identification and naming (Binda et al., 2020). A strain name can be the catalog number of a well-known culture collection or a commercial strain name (Binda et al., 2020). The importance of identification to strain level is becoming increasingly recognized since probiotic health benefits are strain specific, unless otherwise proven (Klein et al., 2010; McFarland et al., 2018). Given the strain specificity of probiotic health benefits, the Joint Food and Agriculture Organization of the United Nations and World Health Organization Working Group (FAO/WHO, 2002) and The Council for Responsible Nutrition and the International Probiotics Association (Council-For-Responsible-Nutrition-and-International-Probiotics-Association, 2017) recommended that strain designation to be described on the labels of probiotic products. However, the molecular basis of a “strain” has not been well defined. A recent review by a probiotic expert panel suggested that strains are defined by a single genetic sequence, and that they can be distinguished by even single nucleotide polymorphisms (SNP) (Jackson et al., 2019). Thus, reliable and highly specific strain identification methodologies are an important component in probiotic authentication and quality assessment.

Methodologies have been developed for the identification of several probiotic species and strains (Solano-Aguilar et al., 2008; Ahlroos and Tynkkynen, 2009; Achilleos and Berthier, 2013; Herbel et al., 2013; Morovic et al., 2016; Shehata et al., 2020; Shehata and Newmaster, 2020a). These methods are conventional PCR or real-time PCR (quantitative PCR, qPCR) based methods. qPCR based methods are widely used in diagnostics because they are fast, sensitive, accurate, allow real time monitoring of reactions, and eliminate the need for post-PCR processing (Wilhelm and Pingoud, 2003). However, designing strain specific qPCR assays can be challenging especially when the target strain belongs to a highly isogenic taxon such as *Bifidobacterium animalis* subsp. *lactis* (Milani et al., 2013). One approach to improve reaction specificity is the use of locked nucleic acids (LNA) since LNA anneal to complementary DNA sequences with higher thermal stability and enhanced selectivity (Singh et al., 1998). LNA assays have been used in previous studies to allow for specificity down to one base pair mismatch (Singh et al., 1998; Johnson et al., 2004), however, to our knowledge it has never been used to help identify probiotics.

The objective of this study was to develop and validate qPCR methods for two clinically important probiotic strains; strain *Bifidobacterium animalis* subsp. *lactis* DSM 15954 and strain *Bifidobacterium animalis* subsp. *lactis* Bi-07™ (Bi-07). DSM 15954 has several health benefits including managing infant colic (Nocerino et al., 2020), a role in reducing the risk of respiratory tract infections in early childhood (Taipale et al., 2016), and a role in improving the periodontal status (plaque index and gingival index) in healthy adults when administered orally as lozenges with *L. rhamnosus* GG (Toiviainen et al., 2015). Strain *Bifidobacterium animalis* subsp. *lactis* Bi-07 was found to reduce the incidence and duration of cold and influenza symptoms (fever, cough incidence, and rhinorrhea duration) in healthy children (Leyer et al., 2009), to improve phagocytic activity of granulocytes, thus improving the immune system functions in healthy elderly adults (Lehtinen et al., 2012), to have immunomodulatory effects in healthy adults (Childs et al., 2014), to contribute to increased lactose digestion in individuals with lactose maldigestion (Turck et al., 2020), and to reduce bacterial translocation and microinflammation in uremic rats (Wei et al., 2014). Therefore, we designed assays incorporating LNA technology for these two clinically important strains.

## Materials and Methods

### Reference Probiotic Strains and DNA Extraction

A total of 13 reference samples of *Bifidobacterium animalis* subsp. *lactis* DSM 15954, 25 reference samples of *Bifidobacterium animalis* subsp. *lactis* Bi-07, and 22 non-target reference samples belonging to other probiotic species were obtained from International Flavors and Fragrances (previously DuPont Nutrition and Biosciences), Nature’s Way Brands, Nature’s Bounty, Jamieson Laboratories Ltd., Lallemand Health Solutions and UAS Labs (Tables 1, 2). DNA was extracted from 50 mg of each sample using NucleoSpin Food kit (740945.50, Macherey Nagel, Germany) according to the manufacturer’s instructions. DNA quantification was performed using Qubit 4.0 Fluorometer (Q33238, Life technologies). DNA was then stored in a −20°C freezer until use.

**TABLE 1:** Samples used to evaluate the analytical specificity of *Bifidobacterium animalis* subsp. *lactis* DSM 15954 strain-specific assay and results for analytical specificity testing.

Sample ID	Sample type	Strain	Mean Cq^#^
1	Target (Mono-strain)	*Bifidobacterium animalis* subsp. *lactis* DSM 15954	22.05
2	Target (Mono-strain)	*Bifidobacterium animalis* subsp. *lactis* DSM 15954	23.19
3	Target (Mono-strain)	*Bifidobacterium animalis* subsp. *lactis* DSM 15954	23.99
4	Target (Mono-strain)	*Bifidobacterium animalis* subsp. *lactis* DSM 15954	22.74
5	Target (Mono-strain)	*Bifidobacterium animalis* subsp. *lactis* DSM 15954	22.46
6	Target (Multi-strain)	*Bifidobacterium animalis* subsp. *lactis* DSM 15954 with other ingredients	22.55
7	Target (Multi-strain)	*Bifidobacterium animalis* subsp. *lactis* DSM 15954 with other ingredients	24.90
8	Target (Multi-strain)	*Bifidobacterium animalis* subsp. *lactis* DSM 15954 with other ingredients	24.98
9	Target (Multi-strain)	*Bifidobacterium animalis* subsp. *lactis* DSM 15954 with other ingredients	26.08
10	Target (Multi-strain)	*Bifidobacterium animalis* subsp. *lactis* DSM 15954 with other ingredients	26.40
11	Target (Multi-strain)	*Bifidobacterium animalis* subsp. *lactis* DSM 15954 with other ingredients	27.05
12	Target (Multi-strain)	*Bifidobacterium animalis* subsp. *lactis* DSM 15954 with other ingredients	26.88
13	Target (Multi-strain)	*Bifidobacterium animalis* subsp. *lactis* DSM 15954 with other ingredients	26.79
14	Non-target	*Bifidobacterium animalis* subsp. *lactis* B420	NA
15	Non-target	*Bifidobacterium animalis* subsp. *lactis* Bi-07	NA
16	Non-target	*Bifidobacterium animalis* subsp. *lactis* Bl-04	NA
17	Non-target	*Bifidobacterium animalis* subsp. *lactis* HN019	NA
18	Non-target	*Bifidobacterium animalis* subsp. *lactis* UABla-12	NA
19	Non-target	*Bifidobacterium animalis* subsp. *lactis* HA-194	NA
20	Non-target	*Bifidobacterium bifidum* Bb-06	NA
21	Non-target	*Bifidobacterium breve* Bb-03	NA
22	Non-target	*Bifidobacterium longum* subsp. *infantis* Bi-26	NA
23	Non-target	*Bifidobacterium longum* Bl-05	NA
24	Non-target	*Lacticaseibacillus casei* Lc-11	NA
25	Non-target	*Lacticaseibacillus* paracasei Lpc-37	NA
26	Non-target	*Lacticaseibacillus rhamnosus* GG	NA
27	Non-target	*Lactiplantibacillus plantarum* Lp-115	NA
28	Non-target	*Levilactobacillus brevis* Lbr-35	NA
29	Non-target	*Ligilactobacillus salivarius* Ls-33	NA
30	Non-target	*Lactobacillus acidophilus* La-14	NA
31	Non-target	*Lactobacillus acidophilus* NCFM	NA
32	Non-target	*Lactobacillus gasseri* Lg-36	NA
33	Non-target	*Limosilactobacillus reuteri* 1E1	NA
34	Non-target	*Lacticaseibacillus rhamnosus* Lr-32	NA
35	Non-target	*Lacticaseibacillus rhamnosus* HN001	NA

*^#^NA means no amplification.*

**TABLE 2:** Samples used to evaluate the analytical specificity of *Bifidobacterium animalis* subsp. *lactis* Bi-07 strain-specific assay and results for analytical specificity testing.

Sample ID	Sample type	Strain	Mean Cq^#^
1	Target (Mono-strain)	*Bifidobacterium animalis* subsp. *lactis* Bi-07	19.19
2	Target (Mono-strain)	*Bifidobacterium animalis* subsp. *lactis* Bi-07	19.69
3	Target (Mono-strain)	*Bifidobacterium animalis* subsp. *lactis* Bi-07	20.23
4	Target (Mono-strain)	*Bifidobacterium animalis* subsp. *lactis* Bi-07	21.40
5	Target (Mono-strain)	*Bifidobacterium animalis* subsp. *lactis* Bi-07 with other ingredients	24.31
6	Target (Multi-strain)	*Bifidobacterium animalis* subsp. *lactis* Bi-07 with other ingredients	24.20
7	Target (Multi-strain)	*Bifidobacterium animalis* subsp. *lactis* Bi-07 with other ingredients	24.27
8	Target (Multi-strain)	*Bifidobacterium animalis* subsp. *lactis* Bi-07 with other ingredients	24.75
9	Target (Multi-strain)	*Bifidobacterium animalis* subsp. *lactis* Bi-07 with other ingredients	24.85
10	Target (Multi-strain)	*Bifidobacterium animalis* subsp. *lactis* Bi-07 with other ingredients	25.61
11	Target (Multi-strain)	*Bifidobacterium animalis* subsp. *lactis* Bi-07 with other ingredients	25.40
12	Target (Multi-strain)	*Bifidobacterium animalis* subsp. *lactis* Bi-07 with other ingredients	25.23
13	Target (Multi-strain)	*Bifidobacterium animalis* subsp. *lactis* Bi-07 with other ingredients	25.54
14	Target (Multi-strain)	*Bifidobacterium animalis* subsp. *lactis* Bi-07 with other ingredients	27.71
15	Target (Multi-strain)	*Bifidobacterium animalis* subsp. *lactis* Bi-07 with other ingredients	27.87
16	Target (Multi-strain)	*Bifidobacterium animalis* subsp. *lactis* Bi-07 with other ingredients	23.52
17	Target (Multi-strain)	*Bifidobacterium animalis* subsp. *lactis* Bi-07 with other ingredients	23.24
18	Target (Multi-strain)	*Bifidobacterium animalis* subsp. *lactis* Bi-07 with other ingredients	24.13
19	Target (Multi-strain)	*Bifidobacterium animalis* subsp. *lactis* Bi-07 with other ingredients	25.04
20	Target (Multi-strain)	*Bifidobacterium animalis* subsp. *lactis* Bi-07 with other ingredients	25.20
21	Target (Multi-strain)	*Bifidobacterium animalis* subsp. *lactis* Bi-07 with other ingredients	25.17
22	Target (Multi-strain)	*Bifidobacterium animalis* subsp. *lactis* Bi-07 with other ingredients	25.11
23	Target (Multi-strain)	*Bifidobacterium animalis* subsp. *lactis* Bi-07 with other ingredients	25.45
24	Target (Multi-strain)	*Bifidobacterium animalis* subsp. *lactis* Bi-07 with other ingredients	26.73
25	Target (Multi-strain)	*Bifidobacterium animalis* subsp. *lactis* Bi-07 with other ingredients	26.38
26	Non-target	*Bifidobacterium animalis* subsp. *lactis* B420	NA
27	Non-target	*Bifidobacterium animalis* subsp. *lactis* BB-12	NA
28	Non-target	*Bifidobacterium animalis* subsp. *lactis* Bl-04	NA
29	Non-target	*Bifidobacterium animalis* subsp. *lactis* HN019	NA
30	Non-target	*Bifidobacterium animalis* subsp. *lactis* UABla-12	NA
31	Non-target	*Bifidobacterium animalis* subsp. *lactis* HA-194	NA
32	Non-target	*Bifidobacterium bifidum* Bb-06	NA
33	Non-target	*Bifidobacterium breve* Bb-03	NA
34	Non-target	*Bifidobacterium longum* subsp. *infantis* Bi-26	NA
35	Non-target	*Bifidobacterium longum* Bl-05	NA
36	Non-target	*Lacticaseibacillus casei* Lc-11	NA
37	Non-target	*Lacticaseibacillus* paracasei Lpc-37	NA
38	Non-target	*Lacticaseibacillus rhamnosus* GG	NA
39	Non-target	*Lactiplantibacillus plantarum* Lp-115	NA
40	Non-target	*Levilactobacillus brevis* Lbr-35	NA
41	Non-target	*Ligilactobacillus salivarius* Ls-33	NA
42	Non-target	*Lactobacillus acidophilus* La-14	NA
43	Non-target	*Lactobacillus acidophilus* NCFM	NA
44	Non-target	*Lactobacillus gasseri* Lg-36	NA
45	Non-target	*Limosilactobacillus reuteri* 1E1	NA
46	Non-target	*Lacticaseibacillus rhamnosus* Lr-32	NA
47	Non-target	*Lacticaseibacillus rhamnosus* HN001	NA

*^#^NA means no amplification.*

### qPCR Assay Design

To design strain-specific qPCR assays, nucleotide variations in the target strain genomes compared to closely related strains should be identified to be targeted in PCR. A novel genome sequence for DSM 15954 was generated using cultured material obtained from German Collection of Microorganisms and Cell Cultures GmbH (DSMZ). The DSM 15954 genome was processed and sequenced as described previously (Banerjee et al., 2021) and was submitted to the National Center for Biotechnology Information (GenBank accession: CP085838, SRA accession: PRJNA773092).

To identify and validate nucleotide variations in the genomes of *Bifidobacterium animalis* subsp. *lactis* DSM 15954 (GenBank: CP085838) and Bi-07 (GenBank: CP003498.1) (Stahl and Barrangou, 2012), the NCBI alignment function and CLC Genomics Workbench 21.0.4 (QIAGEN Bioinformatics) Fixed Ploidy Variant Detection function were used with default parameters. Initially, each strain was aligned to the genome of *Bifidobacterium animalis* subsp. *lactis* Bl-04 (GenBank: CP001515.1). Sequence regions where nucleotide variations were identified were searched on NCBI GenBank against all publicly available sequences to confirm the uniqueness of the identified nucleotide variations to each target strain. Probe-based assays were designed to target the identified nucleotide variations using PrimerQuest Tool (Integrated DNA Technologies (IDT), Coralville, IA, United States). Primers and LNA probes (Table 3) were also ordered from IDT.

**TABLE 3:** *Bifidobacterium animalis* subsp. *lactis* DSM 15954 and *Bifidobacterium animalis* subsp. *lactis* Bi-07 strain specific primer and probe sequences.

*Bifidobacterium animalis* subsp. *lactis* DSM 15954	
Primer F	5′-CATAGATACGACCTCCGTGTG-3′
Primer R	5′-CCGAGAAATCGCTTCACAAC-3′
Probe	5′-ATGCG+A+G+GGCAA-3′ (56-FAM and ZEN – 3IABkFQ)*

***Bifidobacterium animalis* subsp. *lactis* Bi-07**	

Primer F	5′-AACGAGGAGTTGTTCGTATGG-3′
Primer R	5′-GCAGAACCATATTCGCGATTTC-3′
Probe	5′-TCGTGC+C+A+GCG-3′ (56-FAM and ZEN – 3IABkFQ)*

*Locked nucleic acid bases are marked with a + before the base.

### qPCR Protocol

All primers and probes were re-suspended to 100 μM stock solutions (IDT). LNA chemistry was positioned in the probe oligos for both assays (Table 3) as described in previous work in eukaryotes (Johnson et al., 2004). Working solutions of all primers were prepared at 10 μM and working solutions of probes were prepared at 5 μM. Each PCR reaction mixture (20 μl total volume) consisted of 10 μl of 2x SensiFast Probes Master Mix (BIO-86020, Bioline), 4.4 μl of molecular biology grade water, 1.8 μl of each primer (10 μM), 1.0 μl of probe (5 μM), and 1 μl of DNA (DNA concentration is indicated below for the different experiments). PCR running protocol is as follows: denaturation at 95°C for 5 min and amplification (95°C for 10 s, and 66°C for 20 s for *B. animalis* subsp. *lactis* DSM 15954, or 95°C for 10 s, and 64°C for 20 s for *B. animalis* subsp. *lactis* Bi-07) for 40 cycles. Negative no template controls (NTC) were included in each run. All samples in all experiments were tested in triplicate.

### Validation of *B. animalis* subsp. *lactis* DSM 15954 and Bi-07 qPCR Assays

The developed assays were validated following the guidelines for validation of qualitative real-time PCR methods for molecular diagnostic identification of probiotics (Shehata et al., 2019). The assays were validated for specificity, sensitivity, efficiency, repeatability, and reproducibility (Broeders et al., 2014; Shehata et al., 2019). All qPCR reactions were run on QuantStudio 5 Real-Time PCR System (Thermo Fisher Scientific, Mississauga, ON, Canada), except for reproducibility testing which was conducted on both QuantStudio 5 and Hyris bCUBE, a portable qPCR platform.

### Specificity Testing

An essential step in qPCR assay validation is to confirm assay specificity to the target strain. Assay specificity was first evaluated *in silico* by searching the identified unique sequence regions on GenBank using the Basic Local Alignment Search Tool (BLAST) nucleotide function. Assay specificity was also evaluated experimentally. For strain DSM 15954, 13 target and 22 non-target samples (Table 1) were used, and for strain Bi-07, 25 target and 22 non-target samples (Table 2) were used (Shehata et al., 2019). To confirm strain level specificity, closely related *Bifidobacterium animalis* subsp. *lactis* strains (HN019, Bl-04, B420, UABla-12, and HA-194) were included as non-targets for each assay. All samples were tested in qPCR as described above. DNA from all samples was normalized to 1 ng/μl. Each sample was tested in triplicate. True positive rates (ratio of number of correctly classified known positives to total number of known positives) and false positive rates (ratio of number of misclassified known negatives to total number of known negatives) were calculated (Codex-Alimentarius-Commission, 2010; Shehata et al., 2019).

### Sensitivity and Efficiency Testing

Another essential step in qPCR assay validation is to determine assay sensitivity or limit of detection (LOD). Three series of DNA dilutions were used for each target strain. The dilution series were prepared by 10-fold serial dilutions starting from 10, 5, and 2 ng/μl DNA samples. Each dilution series consisted of 5 dilution points (Bustin et al., 2009). Each dilution point was tested in triplicate as described above. To evaluate assay efficiency, standard curves were established between Cq values and log DNA concentration in Prism 9 (GraphPad Software, San Diego, CA, United States). Slope and R square values were determined from the linear regression, and slope values were used to calculate reaction efficiency.

### Precision Testing

To determine precision of the developed assays, repeatability (intraassay variation) and reproducibility (interassay variation) were evaluated. Five target samples at three different DNA concentrations (0.01, 0.1, and 1 ng/μl) were tested in qPCR, on two different days to determine repeatability, and on two different qPCR platforms (bCUBE and QuantStudio 5) to determine reproducibility. Each sample was tested in triplicate.

### Applicability of the Developed Assays for Strain Detection in Finished Dietary Supplements and Food Products

The applicability of the developed assays for strain detection in finished probiotic dosage forms and in food products was evaluated. For each target strain, four samples containing the target strain along with other ingredients commonly used in finished dietary supplements, as well as 12 samples containing the target strains added to various food matrices (Supplementary Tables 1, 2) were tested in qPCR as described above using DNA normalized to 1 ng/μl. Each sample was tested in triplicate.

### Statistical Analysis

Prism 9 (GraphPad Software, San Diego, CA, United States) was used for graphical displays and statistical analyses. Kruskal-Wallis test and Dunn’s multiple comparisons test were used to evaluate the effects of sample matrix on assay performance.

## Results

### qPCR Assay Design

The novel *Bifidobacterium animalis* subsp. *lactis* DSM 15954 genome (BioSample: SAMN22442594) was a single contig 1.94 Mbp in size and was 100% identical to the BB-12 genome (GenBank: CP001853.2) (Jensen et al., 2021) except for several regions: repeated IS2001 family transposases, an intergenic region, and an alpha-glucosidase. Manual inspection of read alignments for each region showed that the polymorphisms were due to errors in repetitive regions, which are notoriously difficult to assemble automatically (Loman et al., 2015), and were manually corrected. Bioinformatic analyses identified a single nucleotide polymorphism (SNP) in each of the genomes of *Bifidobacterium animalis* subsp. *lactis* DSM 15954 and Bi-07 (GenBank: CP003498.1) compared to closely related strains. Two strain specific qPCR assays were designed to target the identified SNPs. The assay for *Bifidobacterium animalis* subsp. *lactis* DSM 15954 amplifies a 135 bp amplicon. The target region codes for a histidine kinase. The assay for *Bifidobacterium animalis* subsp. *lactis* Bi-07 amplifies a 123 bp amplicon, and the target region codes for a glycosyltransferase. Because the assays target a single SNP in each strain, LNA probes were used to enhance assay selectivity.

### Evaluating the Specificity of qPCR Assays

**FIGURE 1:**
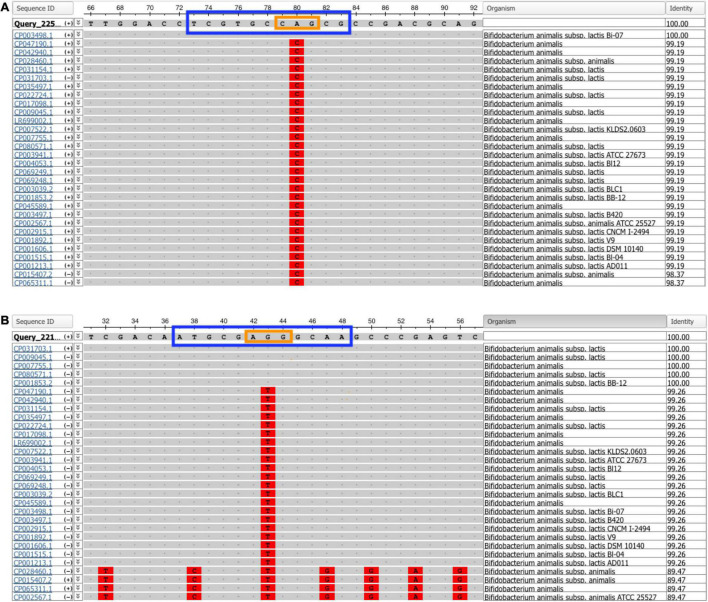
Multiple sequence alignment from NCBI multiple sequence alignment viewer 1.20.1. The amplicon sequence of **(A)** *Bifidobacterium animalis* subsp. *lactis* Bi-07 and **(B)** *B. animalis* subsp. *lactis* DSM 15954 assays were searched on GenBank using the blastn function against the Nucleotide collection database to find matches in all publicly available genome sequences. The probe sequence is in a blue box and the locked nucleic acid bases are in an orange box. The single nucleotide polymorphism (SNP) identified in Bi-07 was unique to strain Bi-07 compared to all other *Bifidobacterium animalis* subsp. *lactis* strains deposited in GenBank, while the SNP identified in DSM 15954 was unique to all strains except IDCC4301, BF052, RH, and i797, as of August 2021.

Specificity of each assay was evaluated *in silico* and experimentally. *In silico* specificity testing revealed that the SNP identified in *Bifidobacterium animalis* subsp. *lactis* Bi-07 is unique to strain Bi-07 compared to all other *Bifidobacterium animalis* subsp. *lactis* strains deposited in GenBank, as of August 2021 (Figure 1A). On the other hand, the SNP identified in *Bifidobacterium animalis* subsp. *lactis* DSM 15954 can differentiate strain DSM 15954 from all other *Bifidobacterium animalis* subsp. *lactis* strains deposited in GenBank, as of August 2021, except for strains IDCC4301, BF052, RH, and i797 (Figure 1B).

**FIGURE 2:**
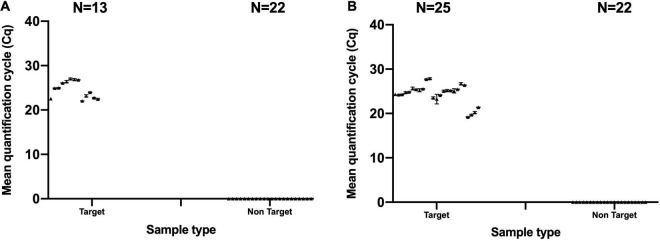
Evaluating the specificity of the developed strain-specific assays. **(A)** *Bifidobacterium animalis* subsp. *lactis* DSM 15954 assay and **(B)** *Bifidobacterium animalis* subsp. *lactis* Bi-07 assay. Numbers of target samples tested were 13 and 25 for DSM 15954 and Bi-07 assays, respectively. Numbers of non-target samples tested was 22 for each assay. Each sample was tested in triplicate.

Evaluating the specificity of *B. animalis* subsp. *lactis* DSM 15954 specific assay in qPCR was conducted using 13 *B. animalis* subsp. *lactis* DSM 15954 target samples and 22 non-target strains. Five out of 13 target samples were mono-strain samples and amplified at a mean Cq between 22.05 and 23.99 and averaged to 22.88 (Figure 2A). Eight out of 13 target samples were multi-strain samples and amplified at a mean Cq between 22.55 and 27.05 and averaged to 25.70 (Figure 2A). None of the non-target samples amplified in this assay, including the closely related *Bifidobacterium animalis* subsp. *lactis* strains (Bi-07, HN019, Bl-04, B420, UABla-12, and HA-194) which confirms strain level specificity (Figure 2A).

Similarly, evaluating the specificity of *B. animalis* subsp. *lactis* Bi-07 specific assay in qPCR was conducted using 25 *B. animalis* subsp. *lactis* Bi-07 target samples and 22 non-target strains. Four out of 25 target samples were mono-strain samples and amplified at a mean Cq between 19.19 and 21.40 and averaged to 20.13 (Figure 2B). Twenty one out of 25 target samples were multi-strain samples and amplified at a mean Cq between 23.24 and 27.87 and averaged to 25.22 (Figure 2B). None of the non-target samples amplified in this assay including the closely related *Bifidobacterium animalis* subsp. *lactis* strains (DSM 15954, HN019, Bl-04, B420, UABla-12, and HA-194) which confirms strain level specificity (Figure 2B). True positive rate for both assays was 100% and false negative and false positive rates were 0%.

### Sensitivity and Efficiency Testing

**FIGURE 3:**
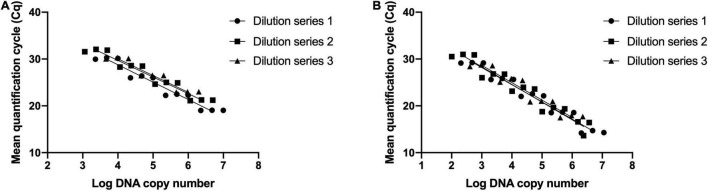
Evaluating the analytical sensitivity and efficiency of the developed strain-specific assays. **(A)** *Bifidobacterium animalis* subsp. *lactis* DSM 15954 assay and **(B)** *Bifidobacterium animalis* subsp. *lactis* Bi-07 assay. Three 10-fold dilution series were prepared from three starting DNA concentrations (10 ng/μl, 5 ng/μl, and 2 ng/μl). Each dilution series was prepared at five dilution points and each dilution was tested in triplicate. Limits of detection were 5 pg, corresponding to 2388 target copies, and 0.5 pg, corresponding to 239 target copies, for DSM 15954 and Bi-07 assays, respectively.

Three DNA dilution series prepared by 10-fold serial dilutions were used to determine limits of detection and reaction efficiency. Standard curves were established for *B. animalis* subsp. *lactis* DSM 15954 assay with slope values of -3.71, -3.55, and -3.59 and reaction efficiency values were 86, 91, and 90% (Figure 3A). R square values were 0.997, 0.998 and 0.999. LOD was determined to be 5 pg, corresponding to 2388 target copies. Standard curves were established for *B. animalis* subsp. *lactis* Bi-07 assay with slope values of -3.67, -3.77, and -3.71 and reaction efficiency values were 87, 84 and 86% (Figure 3B). R square values were 0.998, 0.982 and 0.993. LOD was determined to be 0.5 pg, corresponding to 239 target copies.

### Precision Testing

**FIGURE 4:**
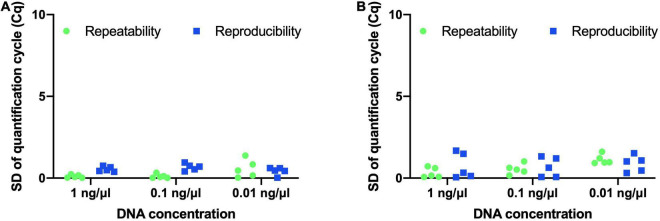
Evaluating repeatability and reproducibility of the developed strain-specific assays. **(A)** *Bifidobacterium animalis* subsp. *lactis* DSM 15954 assay and **(B)** *Bifidobacterium animalis* subsp. *lactis* Bi-07 assay. Five samples at three different DNA concentrations (1 ng/μl, 0.1 ng/μl and 0.01 ng/μl) were used. Each assay was repeated on a different day to evaluate repeatability and was repeated on a different real-time PCR platform (bCUBE and QuantStudio 5) to evaluate reproducibility.

Precision of both assays was evaluated by determining repeatability and reproducibility. *B. animalis* subsp. *lactis* DSM 15954 assay was repeated over a short period of time to determine repeatability. Standard deviation of Cq values between the two trials ranged from 0.02 to 0.24 for 5 samples tested at 1 ng/μl, ranged from 0.01 to 0.33 for 5 samples tested at 0.1 ng/μl, and ranged from 0.03 to 1.38 for 5 samples tested at 0.01 ng/μl (Figure 4A). The *B. animalis* subsp. *lactis* DSM 15954 assay was repeated on two different qPCR platforms (Hyris bCUBE and QuantStudio 5) to determine reproducibility. The standard deviation of Cq values between the two trials ranged from 0.39 to 0.75 for 5 samples tested at 1 ng/μl, ranged from 0.41 to 0.95 for 5 samples tested at 0.1 ng/μl, and ranged from 0.03 to 0.61 for 5 samples tested at 0.01 ng/μl (Figure 4A).

Similarly, the *B. animalis* subsp. *lactis* Bi-07 assay was repeated over a short period of time to determine repeatability. Standard deviation of Cq values between the two replicates ranged from 0.07 to 0.72 for 5 samples tested at 1 ng/μl, ranged from 0.16 to 1.02 for 5 samples tested at 0.1 ng/μl, and ranged from 0.92 to 1.61 for 5 samples tested at 0.01 ng/μl (Figure 4B). The assay was repeated on two different qPCR platforms (Hyris bCUBE and QuantStudio 5) to determine reproducibility. Standard deviation of Cq values between the two replicates ranged from 0.05 to 1.68 for 5 samples tested at 1 ng/μl, ranged from 0.07 to 1.33 for 5 samples tested at 0.1 ng/μl, and ranged from 0.31 to 1.52 for 5 samples tested at 0.01 ng/μl (Figure 4B).

### Applicability of the Developed Assay for Finished Pharmaceutical Products and Food Products

**FIGURE 5:**
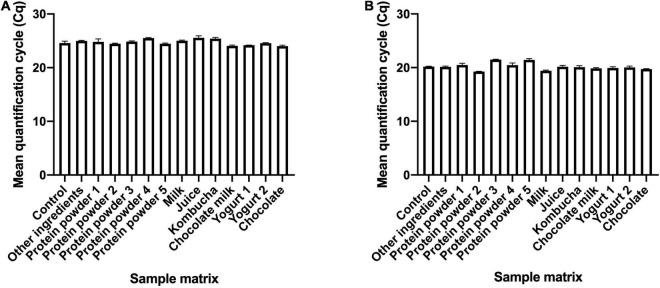
Application of the developed strain-specific assays in various product matrices. *Bifidobacterium animalis* subsp. *lactis* DSM 15954 **(A)** and Bi-07 **(B)** were added to a variety of products and food matrices before DNA extraction. The Cq values from samples with matrices were compared to the Cq value of the culture only control to assess possible PCR inhibition. Shown are bars representing the mean with standard error of the mean (SEM). No significant difference in Cq values was observed between the control sample and any of the sample matrices in both assays (Dunn’s multiple comparisons test), indicating no inhibitory effect.

To evaluate the applicability of the developed assays for use with finished probiotic dosage forms and with food products, four samples containing the target strain along with other ingredients commonly used in finished dietary supplements, and 12 samples containing the target strains added to food matrices were tested using the developed assays. In *B. animalis* subsp. *lactis* DSM 15954 assay, all 16 samples amplified at Cq values ranging from 22.55 to 26.79 (Supplementary Table 1). In *B. animalis* subsp. *lactis* Bi-07 assay, all 16 samples amplified at Cq values ranging from 19.25 to 25.04 (Supplementary Table 2). Food matrices not inoculated with target strains were tested as negative controls for each strain-specific assay, and no amplification was observed from any of the food matrices with both assays. To evaluate the inhibitory effect of other ingredients or food matrices on assay performance, 13 samples that have the same strain composition were compared to a control sample with no ingredients or food matrix. Kruskal-Wallis testing showed significant differences in Cq values (*P*-value = 0.0055 and 0.0049 for DSM 15954 and Bi-07 assays, respectively. However, Dunn’s multiple comparisons test showed that there was no significant difference in Cq values between the control sample and any of the sample matrices in both assays, indicating no inhibitory effect (Figure 5).

## Discussion

*B. animalis* subsp. *lactis* DSM 15954 and Bi-07 are two important probiotic strains with potential beneficial effects to human health. Generally, probiotic products are quantified using culture plating techniques, which are typically species- or genus-specific (Hansen et al., 2020). Although the definition of a strain is not well defined, health benefits of probiotics are considered strain specific unless evidence to prove otherwise exists (Klein et al., 2010; McFarland et al., 2018). Although not all SNPs will have a phenotypic effect, SNPs could result in a protein mutation or a premature stop codon if located in a coding region. Additionally, intergenic SNPs could affect transcription rates, which could lead to phenotypic effects such as antibiotic resistance (Morovic et al., 2018). The probiotic industry is investigating how small changes in probiotic strains like SNPs could affect clinical health benefits, as well as what defines a “strain” (Jackson et al., 2019). The objective of the current study was to develop reliable methods for the specific identification of these two strains for use in laboratory or clinical research as well as in diagnostics to facilitate quality control in commercial probiotic products. Genome sequencing is imperative to establish targets for assay development, which, due to the monophyletic basis of *Bifidobacterium animalis* susp. *lactis*, were only single nucleotide base pairs for this study. LNA oligos are DNA and RNA analogs with increased affinity to enhance PCR assay selectivity that have been widely used for genome-wide association studies in eukaryotes (Johnson et al., 2004). Therefore, LNA oligos were implemented in hydrolysis probe-based qPCR methods for simple, fast, and sensitive identification of the two *B. animalis* subsp. *lactis* strains. To design these strain specific assays, the target genomes were compared to closely related strains to find target SNPs and assays were then validated following the guidelines to determine assay specificity, sensitivity, efficiency and precision (Shehata et al., 2019).

Specificity in targeted qPCR is of paramount importance to confirm that an assay is capable of detecting its target sequence and to eliminate the possibility of amplification and false positive results from non-target strains that are closely related to the target strain (Bustin et al., 2009). Specificity was first evaluated *in silico*, which showed that the SNP targeted in *Bifidobacterium animalis* subsp. *lactis* Bi-07 strain specific assay is unique to strain Bi-07 compared to all other *Bifidobacterium animalis* subsp. *lactis* strains. Similarly, the SNP targeted in *Bifidobacterium animalis* subsp. *lactis* DSM 15954 strain specific assay can differentiate strain DSM 15954 from all other *Bifidobacterium animalis* subsp. *lactis* strains deposited in GenBank except strains IDCC4301, BF052, RH and i797. Further bioinformatic analyses and attempts to target a second region in the DSM 15954 genome to exclude these four strains revealed that there was no single SNP that could exclude all four strains. Frequent updates in sequence databases with frequent depositions of new sequences may necessitate developing additional PCR assays or adding additional targets to ensure strain level specificity. Specificity was also evaluated experimentally for each strain using various related probiotic species and strains (Tables 1, 2). Remarkably, the ratio of number of correctly classified known positives to total number of known positives (True positive rate) was 100% for both assays. The ratio of number of misclassified known negatives to total number of known negatives (false positive rate) was 0% (Codex-Alimentarius-Commission, 2010; Shehata et al., 2019). This shows that highly identical probiotic strains can be distinguished based on a single base pair difference. However, as more commercial strains of the same species become available, new qPCR assays may need to be developed for strain designation.

Another important step in assay validation is to evaluate assay precision or technical variation by determining repeatability and reproducibility. Repeatability measures the agreement of results when an assay is repeated independently under the same conditions over a short period of time (Kralik and Ricchi, 2017). Repeatability was determined for each assay using five samples tested at three concentrations and standard deviation of Cq values between the two replicates was always below 1.38 for *B. animalis* subsp. *lactis* DSM 15954 assay (Figure 4A) and was always below 1.61 for *B. animalis* subsp. *lactis* Bi-07 assay (Figure 4B). The results indicate high precision and minimal intraassay variation. Reproducibility measures the agreement of results when an assay is repeated under different laboratory conditions (Kralik and Ricchi, 2017). The assays performed well on both the standard QuantStudio 5 and the portable bCUBE. The portability of bCUBE facilitates on-site testing under laboratory or non-laboratory settings. These assays can be further optimized and validated as quantitative methods for viable count determination in qPCR or droplet digital PCR based methods (Hansen et al., 2018, 2020; Shehata and Newmaster, 2021). This will require the use of viability dyes to distinguish live versus dead cells, and previous work showed that viability dyes must be optimized for each assay (Kiefer et al., 2020). After generating standard curves, colony forming units (CFUs) can be interpolated based on Cq values.

The developed assays also showed high efficiency and sensitivity. Efficiency is defined as the percentage of target molecules that are copied in a single PCR cycle (Lalam, 2006; Svec et al., 2015). Hence, reaction efficiency is equal to 100% if all target molecules duplicate every cycle. The most reliable method to determine assay efficiency is by constructing standard curves (Bustin et al., 2009; Svec et al., 2015). Reaction efficiency is then calculated from the slope of the curve. To determine assay efficiency, standard curves were established for *B. animalis* subsp. *lactis* DSM 15954 assay and *B. animalis* subsp. *lactis* Bi-07 assays using three DNA dilution series for each strain. Reaction efficiency values for both assays were in the acceptable reaction efficiency range for a qualitative real-time PCR assay, which ranges from 80 to 120% (Broeders et al., 2014). Sensitivity of an assay is the minimum amount of target that can be detected by the assay, and is commonly expressed as the LOD (Bustin et al., 2009). The LOD was determined to be 5 pg for *B. animalis* subsp. *lactis* DSM 15954 assay and was 0.5 pg for *B. animalis* subsp. *lactis* Bi-07 assay. Given the low LOD, the assays are considered highly sensitive, which is advantageous when detecting target strains that exist at low levels in multi-strain products.

To evaluate the applicability of the assays to blends of multiple ingredients, which is common in finished dietary supplement dosage forms, multi-strain samples were tested in qPCR and all samples amplified at a mean Cq value that ranged from 22.55 and 27.05 in *B. animalis* subsp. *lactis* DSM 15954 assay (Figure 2A and Table 1), and a mean Cq value that ranged from 23.24 and 27.87 in *B. animalis* subsp. *lactis* Bi-07 assay (Figure 2B and Table 2). This is an improvement over the standard error that is typical in plate counting. The assays are applicable to both mono-strain samples as well as multi-strain samples and are hence applicable for single ingredient and finished format identification. Furthermore, the matrix effect of food and pharmaceutical ingredients on the assay performance was evaluated to assess the applicability of the assays for food products and for probiotic products formulated with other ingredients such as in finished pharmaceutical forms. All samples added to food or mixed with ingredients successfully amplified (Supplementary Tables 1, 2). Past research showed that various food ingredients can have an inhibitory on PCR amplification (Rossen et al., 1992), which highlights the requirement to validate assays whenever a new food matrix is used. For both assays, no PCR inhibitory effect was observed (Figure 5).

*B. animalis* subsp. *lactis* is widely used in the food and probiotic industry for its health benefits (Milani et al., 2013). However, this taxon is high isogenic nature which makes strain identification a challenge for industry using *B. animalis* subsp. *lactis* strains in their products. Previous studies that investigated compliance in probiotic products could not distinguish between *B. animalis* subsp. *lactis* strains when multiple strains co-existed in a product (Morovic et al., 2016; Shehata and Newmaster, 2020b). Strain specific identification methods employing LNA technology to distinguish single base pair differences facilitate the authentication of *B. animalis* subsp. *lactis* strains whether in single-strain or multi-strain products.

## Conclusion

With the rapid growth in probiotic market, availability of strain identification methods is important to facilitate strain level authentication for probiotic researchers and probiotic industry. The assays developed for the specific identification of strains *Bifidobacterium animalis* subsp. *lactis* DSM 15954 and Bi-07 are qPCR based methods that demonstrate high specificity, sensitivity, efficiency and precision. The assays are applicable to mono-strain and multi-strain samples and also applicable to samples in a variety of food matrices or mixed with pharmaceutical ingredients. The assays can be used on a standard qPCR machine such as QuantStudio 5 Real-Time PCR System or on a portable qPCR machine such as bCUBE for on-site testing. Such strain-specific identification methods offering outstanding performance and broad applicability to sample, matrix and machine types are extremely valuable for strain level authentication to support compliance mission in probiotic products and to ensure probiotic efficacy.

## Data Availability Statement

The datasets presented in this study can be found in online repositories. The names of the repository/repositories and accession number(s) can be found in the article/Supplementary Material.

## Author Contributions

HS designed the study, carried out the experiments, analyzed the data, and wrote the manuscript. AK and WM facilitated sample acquisition, provided valuable comments, and edited the manuscript. SN helped design the study, facilitated sample acquisition, and edited the manuscript. All authors read and approved the manuscript.

## Conflict of Interest

AK and WM were employed by IFF Health & Biosciences, International Flavors and Fragrances, Inc., which commercializes *B. animalis* subsp. *lactis* DSM 15954 and *B. animalis* subsp. *lactis* Bi-07. The remaining authors declare that the research was conducted in the absence of any commercial or financial relationships that could be construed as a potential conflict of interest.

## Publisher’s Note

All claims expressed in this article are solely those of the authors and do not necessarily represent those of their affiliated organizations, or those of the publisher, the editors and the reviewers. Any product that may be evaluated in this article, or claim that may be made by its manufacturer, is not guaranteed or endorsed by the publisher.
